# 1.0 T open-configuration magnetic resonance-guided microwave ablation of pig livers in real time

**DOI:** 10.1038/srep13551

**Published:** 2015-08-28

**Authors:** Jun Dong, Liang Zhang, Wang Li, Siyue Mao, Yiqi Wang, Deling Wang, Lujun Shen, Annan Dong, Peihong Wu

**Affiliations:** 1 Department of Medical Imaging & Image Guided Therapy, Sun Yat-Sen University Cancer Center; State Key Laboratory of Oncology in South China; Collaborative Innovation Center for Cancer Medicine; East Dong Feng Road 651, Guangzhou, Guangdong 510060, P. R. China

## Abstract

The current fastest frame rate of each single image slice in MR-guided ablation is 1.3 seconds, which means delayed imaging for human at an average reaction time: 0.33 seconds. The delayed imaging greatly limits the accuracy of puncture and ablation, and results in puncture injury or incomplete ablation. To overcome delayed imaging and obtain real-time imaging, the study was performed using a 1.0-T whole-body open configuration MR scanner in the livers of 10 Wuzhishan pigs. A respiratory-triggered liver matrix array was explored to guide and monitor microwave ablation in real-time. We successfully performed the entire ablation procedure under MR real-time guidance at 0.202 s, the fastest frame rate for each single image slice. The puncture time ranged from 23 min to 3 min. For the pigs, the mean puncture time was shorted to 4.75 minutes and the mean ablation time was 11.25 minutes at power 70 W. The mean length and widths were 4.62 ± 0.24 cm and 2.64 ± 0.13 cm, respectively. No complications or ablation related deaths during or after ablation were observed. In the current study, MR is able to guide microwave ablation like ultrasound in real-time guidance showing great potential for the treatment of liver tumors.

In popular liver cancer clinical guidelines, ablation therapy is the recommended curative treatment modality. Ultrasound and computerized tomography (CT) are the most common methods for these ablation procedures. However, CT exposes the operator to radiation that cannot be utilized for real-time guidance. Ultrasound provides real-time guidance, but the image quality is worse than CT and magnetic resonance imaging (MRI). In contrast, MRI is characterized by high soft-tissue contrast, sensitivity to thermal signals, and multiplanar capabilities^1^. With MRI, we can better visualize tumor tissue and the surrounding anatomy, and obtain three-dimensional visualization of the antenna and the target zone. Perhaps the greatest benefit is that there is no X-ray exposure to either the patient or the interventionalist.

Since Morikawa *et al.* firstly reported microwave ablation as a feasible tool for MR-guided interstitial hyperthermia at the 8th Annual Meeting of International Society for Magnetic Resonance in Medicine (ISMRM) in 2000, studies on microwave ablation of tumors using MR guidance have been developed. However, image interference generated by radiofrequency or microwave devices in ablation limits the utility of MR. Nevertheless, Morikawa *et al.* performed MR-guided microwave ablation of liver tumors using a 0.5 T open MRI system in 2002^2^. In this study, they successfully eliminated the radiofrequency interference during ablation after installation of a notch filter and subsequently obtained clear images^2^. However, the acquisition time for each image was 6.8 s. After that, a series of studies using MR-guided therapy were performed. For instance, Kurumi *et al.* performed MR-guided microwave ablation on 24 patients with liver tumors and shortened the acquisition time for each slice to less than 2 s^3^. Morikawa *et al.* firstly applied respiratory triggering for MR-guided microwave ablation of liver tumors under general anesthesia and overcame the problem of motion artifacts^4^. Abe *et al.* performed MR-guided microwave therapy for metastatic liver tumors from breast cancer using open-configuration MR and displayed near-real-time imaging and thermal images on a monitor^5^. The acquisition time of each slice was still less than 2 s. Fischbach F *et al.* completed magnetic resonance-guided freehand radio-frequency ablation of malignant liver lesions using an interactive open magnetic resonance scan platform. They successfully reduced the time for each single image slice to 1.3 s^6^.

To the best of our knowledge, the latest and fastest frame rate of each single image slice for MRI is 1.3 seconds. According to statistic data, the average reaction time for recognition of movement in human is 0.33 seconds^7^. It means that the frame rate for each slice at 1.3 s cannot show us continuous images, but interrupted and delayed images. Therefore, previous studies did not reach the frame rate for real time imaging, which greatly limits the accuracy of puncture and ablation, and can result in puncture injury or incomplete ablation. For tumors in locations close to vessels or important tissue, delayed images can be particularly dangerous to patients receiving MR-guided ablation. In order to obtain faster frame rate of single image slices and reach real real-time guidance and monitoring, we performed the animal experiments of MR-guided microwave ablation. In the current study, we successfully administrated microwave ablation under real-time MR guidance and monitoring at the frame rate for each single image slice 0.2 s *in vivo*.

## Materials and Methods

### Industry support

**Figure 1:**
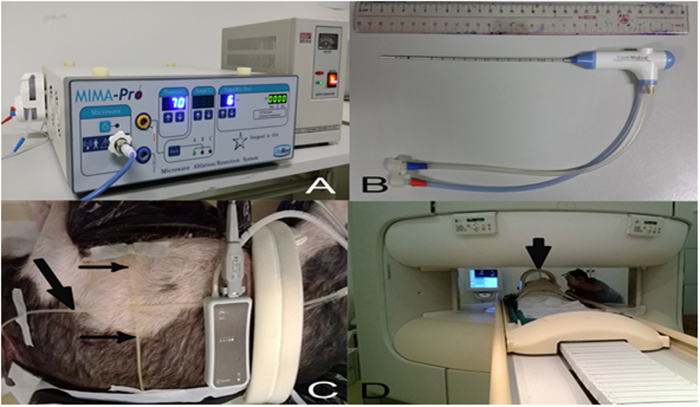
Materials and methods for the experiment. The microwave ablation generator and electric transformer can work in the magnetic resonance (MR) operation room. (**A**) The internally cooled antenna is specially designed for MR-guided microwave ablation (**B**). The diameter of the antenna is 2 mm and the length is 17.5 cm. The oil line (black arrows) designed by us was crossed on the body surface and used as an imaging marker (**C**). At right, the respiratory-triggered device was bound to the abdominal surface. This experiment was performed using 1.0 T whole-body open configuration MR scanner (**D**) using a head and neck coil (black arrow).

Qiya Ltd. Co. (Nanjing, People’s Republic of China) donated the microwave generator (Fig. 1A) and provided the ablation antennas (Fig. 1B) through a materials-transfer agreement with the Qiya/Sun Yat-sen University Cancer Center Cancer Treatment Program. The authors had full control of the data and information submitted for publication.

### Animal care

Under the supervision of the Division of Laboratory Animal Medicine at our institution, 10 Wuzhishan pigs (Da Hua Nong Animal Care Ltd. Co., Guangdong, People’s Republic of China) weighing 17.5–27.2 kg were studied in this study. All animals received appropriate humane care in compliance with guidelines set by the Laboratory Animal Care and Use Committee and Ethics Committee at our institution. All experimental protocols were approved by both Laboratory Animal Care and Use Committee and Ethics Committee of Sun Yat-sen University Cancer Center.

### Preparation before ablation

General anesthesia was induced (15–20 mg/kg 3% pentobarbital; Fatal Plus 0.25 mL/kg; Vortech Pharmaceuticals, Dearborn, MI, USA) by injection into the neck muscles of pigs. If anesthesia did not achieved in 10 minutes, an additional dose 3–6 mg/kg would be injected. For the puncture and ablation procedure, 5 mg/kg pentobarbital solution was injected through the venous indwelling needle in the ear every 90 minutes. Xylazine hydrocloride injection (Shenda Pharmaceuticals, Jilin, People’s Republic of China) was alternatively used during the procedure for fast anesthesia.

A venous indwelling needle in the ear was used for injection of contrast medium or rescue or intravenous anesthesia. Before ablation, pigs were placed on an MRI scan bed under intravenous anesthesia and monitored by an anesthesiologist throughout procedure.

The pig was posed in the left lateral position. Imaging markers (oil line, designed by the surgeons) were crossed on the abdomen surface. A respiratory-triggered device was bound to the abdominal surface where the maximum motion amplitude of breathing movements was obtained (Fig. 1C). The respiratory-triggered device was used for better quality images to eliminate the interference as a result of breathing.

### Scanning sequences

MRI was performed using a 1.0-T whole-body open configuration MR scanner (Panorama HFO; Philips Healthcare, Eindhoven, Netherlands; Fig. 1D). Because of the small body circumference of the animals, a half-open head and neck coil was used for the MRI scan; it was also suitable for both puncture and scan (lower left corner Fig. 1D). A respiratory-triggered liver matrix array was tested, including pre-enhanced axial, sagittal, and coronal T1-weighted (T1W) sequences, axial, sagittal, and coronal T2W sequences, interactive step-by-step sequence for real-time guidance and monitoring, and dynamic contrast-enhanced axial, sagittal, and coronal T1W sequences after ablation. The contrast medium (Gadolinium–DTPA; Magnevist, Bayer Schering Pharma, Berling-Wedding, Germany) was intravenously administered at a dose of 0.2 mmol/kg for dynamic contrast-enhanced images. All images were transferred to the Centricity RIS/PACS workstation (Centricity RIS CE Systems; GE Healthcare, Cleveland, OH, USA) for storage. The interactive scan was designed and tested for real-time guidance and monitoring of the puncture and ablation procedure. The videos of real-time guidance and monitoring procedure were recorded by camera. On the pre-enhanced MR images, signal intensity was classified as hypointense, isointense, or hyperintense. Conversely, on the dynamic contrast-enhanced MR images, the enhancement intensity was classified as none, mild, moderate, or significant enhancement^6^.

### Ablation procedure

#### Design of puncture route

**Figure 2:**
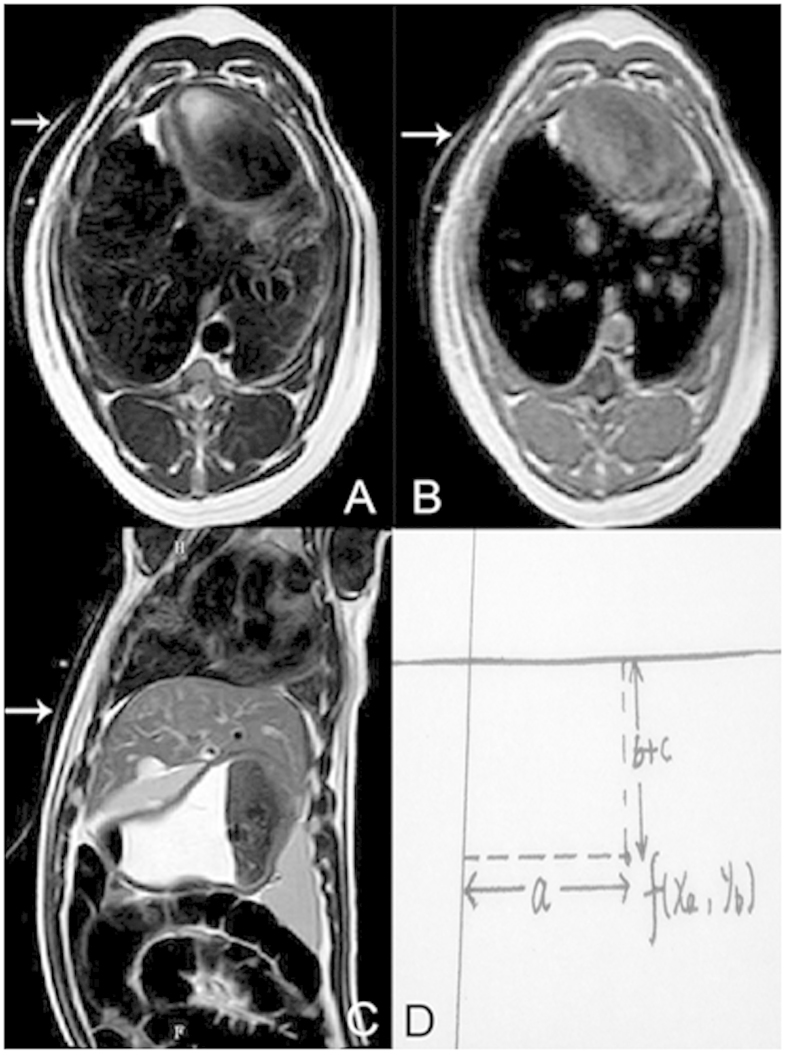
Coordinate method for calculating puncture route. Imaging markers represent a hyperintense line around the surface on the axial T2W image (**A**), T1W image (**B**), and sagittal T2W image (**C**). The formula: f(*x*_*a*_, *y*_*b*_) = (*a*, *b* + *c*), illustrates the process of designing the puncture route (**D**). The *x*-axis represented on the imaging marker is perpendicular to the body line; the *y-*axis represented on the imaging marker runs parallel to the body line; the *a* represents the distance from the *y*-axis; *b* represents the slice thickness used for scanning; and *c* represents the slice gap used for scanning.

After pre-contrast scan, axial, sagittal, and coronal images clearly showed the planning target area for ablation. An appropriate route for puncture and ablation was designed. With the help of the crossing image marker placed on the body surface of the pig, the designed route was calculated using the coordinate method by markers on the surface of the target area (Fig. 2). The angle for puncture was controlled by the size and location of the target area. The formula for the coordinate position was:


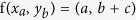


In the formula, the *x*-axis represented the imaging marker perpendicular to the body line, *y-*axis represented the imaging marker parallel to the body line, *a* represented the distance from the *y*-axis, *b* meant the slice thickness, and *c* represented the slice gap used for the scan.

#### Puncture procedure under real-time guidance

**Figure 3:**
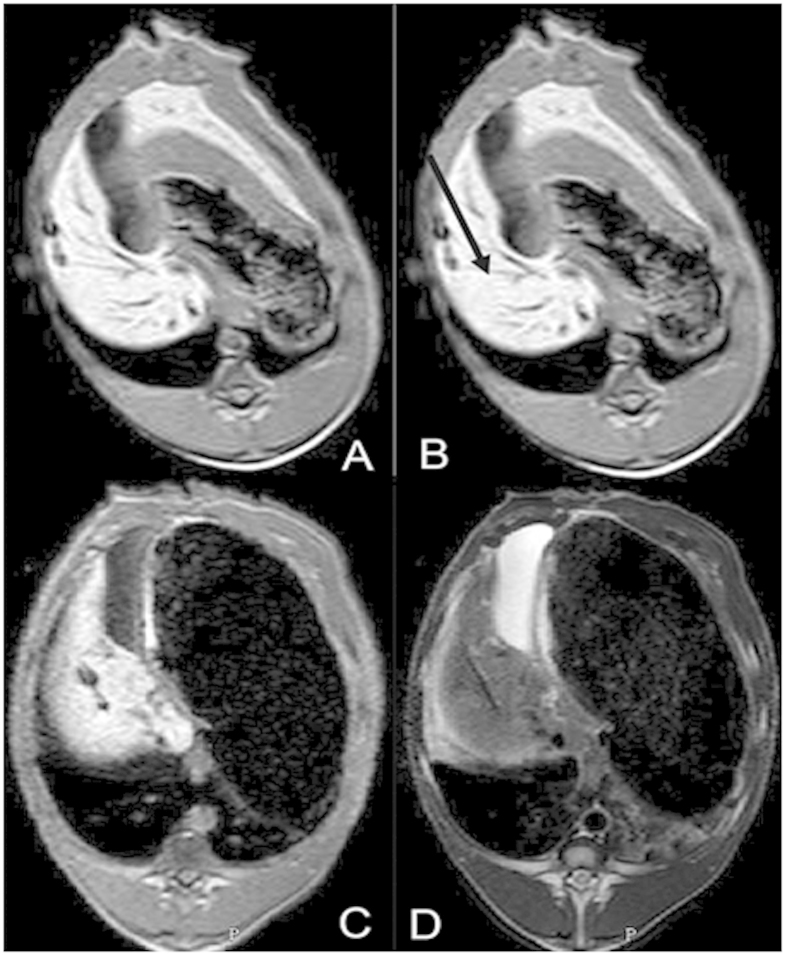
Puncture procedure and adjustment. Another T1W scan was performed to confirm the position of the antenna in the liver after puncture (**A**). Based on the position, the angle and depth of the antenna in the liver and the distance from the target area on T1W images, real-time adjustment of the antenna was performed to make accurate puncture in the target area (**B**). Additional T1W and T2W scans were performed to confirm the position of the antenna (**C**,**D**).

Once the puncture route was designed, the invasion site was also labeled on the surface of the target area of the pig. After skin antisepsis, disinfection, and incision on the designated invasion site, the microwave ablation antenna was inserted into the body of the pig. With the help of continual scanning, we inserted the antenna under real-time guidance of MR via the synchronous monitor in the operating room (Video 1). The frame rate of each single image slice was only 0.202 seconds. On real-time scan images, the antenna was manifested as long hypointense line, easily contradistinguished from liver. We could clearly track the position, angle, and depth of the antenna in the liver and the distance from the target area on the monitor. Real-time adjustment of the antenna was performed to ensure the right position. Additional T1 and T2W scans were sequentially performed to ensure the antenna was positioned in the target area (Fig. 3).

#### Ablation procedure under real-time guidance

When the power for microwave ablation was set according to the location and the size of the target area, the microwave generator initiated. At the same time, real-time continual scans commenced. On the monitor screen, we observed the changes in the ablation area on MR images (Video 2). When the ablated area completely encompassed the target area, the ablation stopped. Additional T1 and T2W plain and contrast scans were performed after ablation to confirm a safe margin around the target area. After that, the ablation antenna was pulled out to end the procedure. Finally, T1 and T2W plain scan was performed once again to check for bleeding or not. If there was an emergency during the procedure, appropriate rescue procedures would be undertaken at this point.

### Adverse events

To dynamically observe adverse events, follow-up for each pig was randomly arranged at 0, 1, 2, 3 or 4 weeks after receiving microwave ablation. Ablation damage was monitored via blood test and imaging. Venous blood was obtained from pigs before and after ablation for routine blood tests, liver function tests, and renal function tests. If there were any deaths, the cause was recorded.

### Statistical analysis

Differences in blood test results before and after ablation were compared using the *t*-test. All statistical tests were 2-sided, and a *P* < 0.05 was defined as statistically significant. The analyses were performed with Statistical Package for the Social Science (SPSS version 16.0; SPSS Inc., Chicago, IL, USA).

## Results

### General information

In total, 10 Wuzhishan pigs were enrolled in the liver microwave ablation experiments; two males and eight females. The mean age of these animals was 234 days and the mean weight was 21.81 kg. Pig details are shown in Table 1.

### Scanning parameters for real-time imaging

During the experiment, we obtained perfect respiratory-triggered liver sequences. The parameters for these sequences were as follows: (1) T1W turbo-field echo (TFE) sequence (repetition time [TR]: 14 ms; echo time [TE] = 6.9 ms; slice thickness, 5.0 mm; slice gap, −0.6 mm; matrix scan, 180 × 135; total scan time: 02:24 minutes); (2) T2W turbo-spin echo (TSE) sequence (TR, 1464; TE, 80 ms; slice thickness, 4.0 mm; slice gap, 0.4 mm; and matrix scan, 244 × 168; total scan time: 02:15 minutes); (3) real-time imaging sequence (TR, 3.1; TE, 1.57 ms; slice thickness, 10.0 mm; slice gap, 1.0 mm; and matrix scan, 96 × 94; total scan time: 0.202 s); and (4) dynamic contrast scan sequence (TR, 3.4; TE, 1.7 ms; matrix scan, 172 × 135; total scan time: 01.23.8 min). With these sequences/parameters, we obtained real-time guidance and monitoring of the entire procedure. The shortest time for frame acquisition was 0.202 s. Details of these procedures are shown in Table 2.

### Ablation results

The mean size of liver samples was 19.24 cm. The first and second pigs underwent a preliminary test to obtain further information of the equipment and MRI sequences. The time for the puncture procedure was defined from the time when the antenna was inserted into the body to the antenna was posed in the planned position; it was 23 minutes and 15 minutes, respectively. The lengths of the ablated, nearly oval areas were 2.78 cm and 2.83 cm using a power of 60 W, respectively; the widths were 1.65 cm and 1.72 cm, respectively. The other eight pigs were operated based on the preliminary tests. The mean puncture time was shorted to 4.75 minutes and the mean ablation time was 11.25 minutes with a power of 70 W in these animals. The mean ablation length and the widths were 4.62 cm and 2.64 cm, respectively. Details from these pigs are shown in Table 3.

### MR representation

#### Plain scan representation

**Figure 4:**
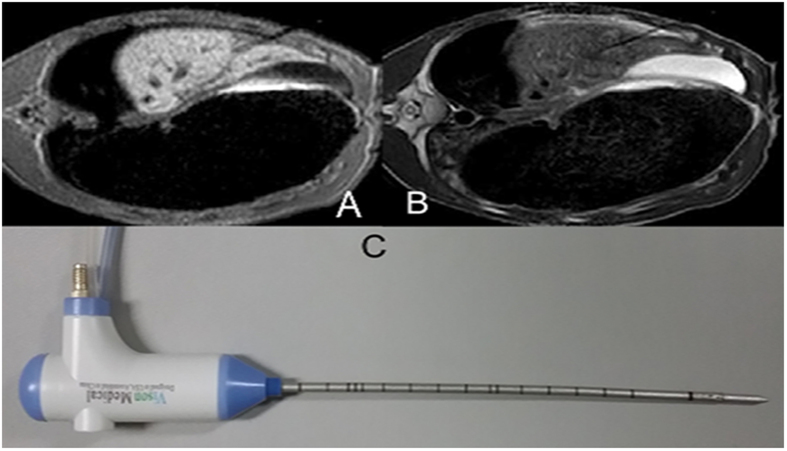
The microwave ablation antenna and representation on T1W and T2W images. The antenna (**C**) represented an apparent widening and blooming ball-shaped signal void at the needle tip on T1W images (**A**). The antenna represented a thin hypointense line on T2W image without any artifacts (**B**).

On T1W plain scan images, the antenna represented a long and apparent widening hypointense single line, which was easily distinguished from the normal liver. There was a blooming ball-shaped signal void at the needle tip. On T2W plain scan images, the antenna represented a long hypointense line without surrounding artifacts. The width of the hypointense line was similar to the antenna (Fig. 4).

#### Real-time scan representation

In the real-time imaging, vascular showed high signal, the liver was gray signal, gallbladder showed high signal, subcutaneous fat showed high signal. In real-time guided puncture, microwave ablation antenna clearly represented strip of black signal, lower than the liver, same width as ablation antenna. The ablation antenna was moving with breathing exercises. Real-time imaging displayed clear and real-time position of the antenna.

In real-time monitoring of the ablation process, the central ablation area showed linear black signal, gradually expanding the scope from ablation antenna. Slightly higher signal area around the black central area with clear boundary representing oval, was gradually expanding. Changes of ablation were clearly showed in the real-time imaging.

#### Representation after ablation

**Figure 5:**
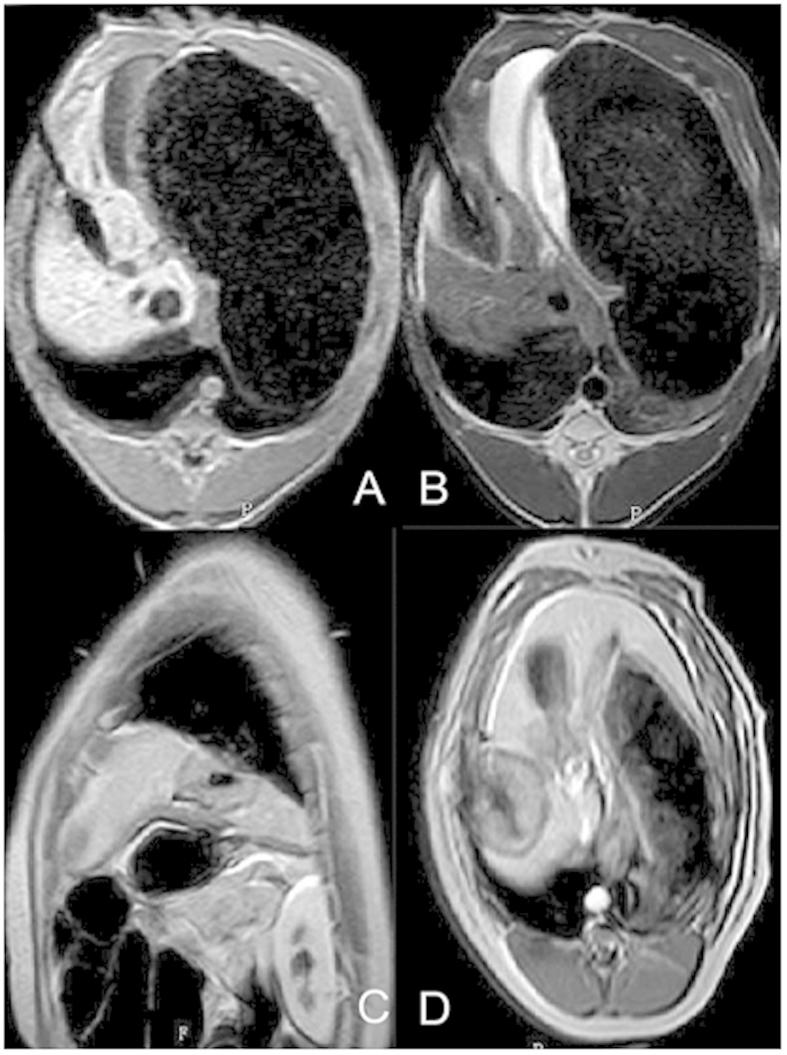
Imaging characteristics after microwave ablation. The ablated area represented a thin oblong hypointense oval around the antenna on T1W images (**A**) and thick oval hypointense with hyperintense surroundings on T2-weighted images (**B**). On contrast-enhanced T1-weighted images, the ablated area is represented by a thin line hypointense in the center, while enhancement of the ablated area was hypointense (**C**,**D**).

Instantly after ablation, central ablation area showed long oval, back and low signal around the antenna. Peripheral ablation area showed slightly hyperintense or isointensity in liver with clear or fuzzy boundary. A hypointensity ring was around the boundary on T1WI (Fig. 5A). Ablation area showed oval and low signal around the antenna on T2WI. A hyperintensity ring was around the hypointensity (Fig. 5B). Ablation area showed large oval and gray signal around the antenna on contrast-enhanced images. A hypointensity ring was around the ablation area. Another hyperintensity ring was outside, mapping the extent consistent with the ablation zone in T2WI (Fig. 5C,D).

### Complications and safety

Of the 10 pigs, no deaths occurred immediately after the ablation procedure; nine pigs were euthanized as planned. Only one pig died of asphyxia during transportation back to the animal facility after ablation. There were no serious complications, and ablation related deaths. Bleeding during the puncture was the most common complication; however, the mean blood loss during the entire procedure was only 6.3 mL. There were no differences in main liver function or renal function before and after ablation (*P* > 0.05). However, gamma-glutamyl transpeptidase (GGT) and aspartate transaminase (AST) were significantly higher after ablation (*P* < 0.05). There were no differences in hemoglobin, red blood cell, or albumin counts. Other complications were not monitored. Details of these complications are shown in Table 4.

## Discussion

In the current study, we successfully administered microwave ablation in pig livers under MR real-time guidance and monitoring in 10 pigs. Considering that MR works using radiofrequency for imaging, the interference would be serious if we chose radiofrequency ablation. In addition, previous study did not detailedly show how to eliminate the interference generated when radiofrequency ablation undergoing^6^. This is the reason for we chose microwave ablation rather than radio-frequency ablation. The mean age of these animals was 234 days and the mean weight was 21.81 kg. Although these pigs are small, they are large enough for liver ablation. Moreover, too old or too big pigs render the operation more complicated. We followed up these pigs at 1 week, 2 weeks, 3 weeks, and 4 weeks to perform blood tests and obtain liver samples. Only one pig died of asphyxiation during transport back to the animal facility after ablation.

During the procedure, in order to obtain optimal image quality and reduce interference caused by the animals’ breathing, a respiratory-triggered scan array was applied. Because of interactions with MR machine when microwave generator was working, there was noise signal that blocked the real-time imaging. To utilize real-time guidance and monitoring, we should firstly eliminate interferences of microwave generator. Finally, we redesigned and added insulation device to the microwave generator. After several iterations, we eventually determined the optimal scan parameters for real-time guidance by balancing scan time and image quality after resolving the problem of noise signal.

In the pre-operation plain scans, T1W-TFE and T2W-TSE were utilized for locating tumor position and designing puncture route. Because of a blooming ball-shaped artifacts at the antenna tip, T1W-TFE is not suitable for guidance or locating. The antenna represented clearly on T2W-TSE without artifacts around the tip. So it is suitable for locating the antenna.

After locating, an interactive step-by-step sequence is designed for real-time guidance and monitoring. To reach real-time guidance and monitoring, we firstly ensure fast and continuous image acquisition. Imaging speed and qualities should be well balanced. After several tests, we finally decided the best parameters for real-time scanning. The overall time for acquiring a single image was only 0.202 s. A study published in 2010 found that athletes had an average reaction time of 0.203 seconds, while the recognition of movement was not less than 0.33 seconds^8^,^9^. This means that those moving images are perceived to be continuous in human eyes, as is the case of ultrasound. There is no pause in the continuous images, like a movie or video. In this sense, microwave ablation was performed under MR real real-time guidance for the first time. In the real-time imaging, it is different from the T1WI or T2WI. The blood vessel showed high signal, the liver was gray signal, gallbladder showed high signal, subcutaneous fat showed high signal. The microwave antenna clearly represented strip, black signal, as width as the antenna. Real-time imaging displayed clear and real-time position of the antenna. With the help of real-time imaging, the puncture process could be guided under our eyes. Especially for those lesions closing to complex anatomy or large blood vessels, we could clearly map the tip and the distance from vessels to ensure the safety. Further more, we also eliminate blind spots by any angle imaging in real-time imaging. After puncture, we also get real-time monitoring the ablation process. The central ablation area showed linear black signal, gradually expanding the scope from ablation antenna. Slightly higher signal area around the black central area with clear boundary representing oval, was gradually expanding. Changes of ablation were clearly showed in the real-time imaging. When the boundary covered the planed area, the ablation stopped. If the is any residual lesion, additional ablation would be given. We could ensure the treatment effects of ablation by real-time monitoring ablation extent. On the other hand, it also makes ablation safe from damaging surrounding important anatomy, large blood vessel, gallbladder etc.

After ablation, the ablated area represented a thin, oval hypointense area around the antenna on T1W images, and a thick oval hypointense with surrounding hyperintense area on T2W images. On contrast-enhanced T1W images, the ablated area represented a thin hypointense line in the center, with enhancement of the ablated area and hypointense surrounding area (Fig. 5). T2W and contrast-enhanced T1W clearly mapped the ablated area with distinct boundaries, while T1W was not suitable for mapping the ablated area.

With these sequences, under MR real-time guidance, the puncture time was reduced to 3 minutes with experience. The ablation time ranged from 5 minutes to 15 minutes, with the mean time was 11.25 minutes. Measurements of liver samples after ablation showed a mean width of 2.64 ± 0.13 cm and a mean length of 4.62 ± 0.24 cm, which are consistent with previous reports^10^,^11^. Yu *et al.* reported that after 10 minutes of ablation, the short-axis diameter and the long-axis diameter following 2,450 MHz microwave were 2.35 ± 0.75 cm and 3.86 ± 0.81 cm, respectively^10^. Similarly, Lubner *et al.* also reported that, for single antennas, mean ablation zone lengths were 3.5 ± 0.55 cm and 4.2 ± 0.40 cm at 5 and 10 minutes, respectively; mean widths were 2.0 cm ± 0.32 and 2.5 cm ± 0.25 at 5 and 10 minutes, respectively^12^.

At last, we showed that MR guided microwave liver ablation was safe. There is no serious complications or ablation-related deaths. A small amount of bleeding during the puncture was the most common minor complication. Moreover, there were no differences in liver function, renal function, or routine blood tests before or after ablation (*P* > 0.05).

In conclusion, in this study, we successfully performed MR-guided microwave ablation under real real-time guidance and monitoring, and described the detailed procedure and image characteristics. In this preliminary experiment, we show that MR-guided microwave ablation is a safe and effective treatment without X-ray exposure. It can be performed in real-time with high soft-tissue contrast providing better visualization of tumor tissue and surrounding anatomy. We believe that real-time monitoring throughout the ablation procedure with three-dimensional visualization of the antenna and the target zone will become the trend for tumor therapy.

## Additional Information

**How to cite this article**: Dong, J. *et al.* 1.0 T open-configuration magnetic resonance-guided microwave ablation of pig livers in real time. *Sci. Rep.* **5**, 13551; doi: 10.1038/srep13551 (2015).

## Supplementary Material

Supplementary Video 1

Supplementary Video 2

Supplementary Information

## Figures and Tables

**Table 1:** General characteristics of experimental animals.

Number	Sex	Age (days)	Weight (kg)	Experiment date	Execute date
1	F	180	20.5	2014-1-12	2014-2-16
2	M	300	26.8	2014-1-19	2014-2-16
3	F	300	27.2	2014-3-2	2014-3-3
4	F	300	26.3	2014-3-3	2014-3-16
5	F	210	17.5	2014-3-10	2014-3-24
6	F	210	17.6	2014-3-10	2014-3-23
7	M	210	22.6	2014-4-8	2014-4-13
8	F	210	23.2	2014-4-5	2014-4-27
9	F	210	18.5	2014-4-6	2014-5-3
10	F	210	17.9	2014-4-6	2014-5-3

**Table 2:** Parameters of the imaging scans.

Sequences	TR (ms)	TE (ms)	Matrix scan	Axial slice thickness (mm)	Axial slice gap (mm)	Coronal and sagittal slice thickness (mm)	Coronal and sagittal slice gap (mm)	Overall time(min)	Scan time/resp (min)
T1W-TFE	14	6.9	180*135	5	−0.6	5	−0.6	02:24.0	00:01.3
T2W-TSE	1464	80	244*168	4	0.4	6	0.6	02:15.0	00:01.5
Interactive step-by-step	3.1	1.57	96*94	10	1	10	1	00.00.202	—
Dynamic contrast-enhanced scan	3.4	1.7	172*135	—	—	—	—	01:23.8	—

T1W-TFE: T1-weighted turbo-field echo; T2W-TSE: T2-weighted turbo-spin echo; TR: repetition time; TE: echo time; ms: millisecond; mm: millimeter; min: minute.

**Table 3:** General parameters/characteristics of the equipment and ablation results.

No.	Power (W)	Frequecy (MHz)	Ablation location	Liver size (cm)	Puncture time (min)	Ablation time (min)	Ablated area length (cm)	Ablated area width (cm)
1	60	2450	Liver	19.2	23	5	2.78	1.65
2	60	2450	Liver	18.3	15	5	2.83	1.72
3	70	2450	Liver	17.8	10	10	4.62	2.56
4	70	2450	Liver	15.6	5	5	3.20	2.17
5	70	2450	Liver	22.5	5	15	5.12	2.78
6	70	2450	Liver	15.5	5	10	4.67	2.54
7	70	2450	Liver	21.3	4	10	4.58	3.05
8	70	2450	Liver	21.9	3	15	5.32	2.94
9	70	2450	Liver	20.7	3	15	5.25	2.98
10	70	2450	Liver	19.6	3	10	4.23	2.12

W: watt; MHz: megahertz; cm: centimeter; min: minute.

**Table 4:** Results of blood tests before and after the ablation experiment.

Complications	Before ablation	After ablation	*P*
ALT	60.28	65.39	0.588
AST	34.45	90.15	0.002
GGT	145.88	165.58	0.013
ALP	68.48	57.79	0.495
Total serum protein	64.59	69.04	0.369
Albumin	36.27	32.62	0.486
Total bilirubin	1.38	1.97	0.626
Direct bilirubin	0.87	1.14	0.768
Blood urea nitrogen	3.036	3.312	0.116
Blood urea nitrogen	62.87	55.9	0.753
RBC	7.9	8.048	0.346
Hb	133.2	137.9	0.483
PLT	402.7	355.2	0.244

ALT: Alanine aminotransferase; AST: aspartate aminotransferase; GGT: glutamyl transpeptidase; RBC: red blood cells; Hb: hemoglobin; PLT, platelets.
