# Supplementary material for: 1.0 T open-configuration magnetic resonance-guided microwave ablation of pig livers in real time
Source: Sci Rep. 2015 Aug 28;5:13551. doi: 10.1038/srep13551 (PMC4551954; doi:10.1038/srep13551)
Supplement: Supplementary Information [file srep13551-s3.pdf]

1.0 T open-configuration magnetic resonance-guided microwave ablation of pig livers in real time

Running title: MR-guided microwave ablation in real-time

Jun Dong[1▲], Liang Zhang[1▲], Wang Li[1], Siyue Mao[1], Yiqi Wang[1], Deling Wang[1], Lujun Shen[1], Annan Dong[1], Peihong Wu[1*]

Video legends

Movie 1:

Real-time monitoring puncture procedure using MR as like ultrasound. In the movie, with help of the interactive sequence and respiratory trigger technology, we could clearly see smooth and continuous images of whole puncture procedure as ultrasound displays. The microwave ablation probe in the liver presents a linear hypointense distinguished from pig normal liver tissue, which shows a slight hyperintensity on images. The images are characterized by high soft tissue contrast and there is no probe thickening and shadow. The most important point is that what you are doing is completely synchronous displaying in the procedure using MR.（This video was generated by the interactive step-by-step sequence (TR, 3.1; TE, 1.57 ms; slice thickness, 10.0 mm; slice gap, 1.0 mm; and matrix scan, 96 × 94; total scan time: 0.202 s) and taken from monitor screen of MR by Nokia photo capture software）

Movie 2:

Real-time monitoring ablation procedure using MR as like ultrasound. In the smooth and continuous images, the whole ablation procedure in liver is displayed in front of us without any interfere. When ablation started, we can see an oval hyperintensity ring spreading around in the liver from the probe. In the center of the hyperintensity ring, increasing even hypointensity is detected. The oval hyperintensity ring indicates the ablated area, which means we could map the ablated area through real-time monitoring and avoiding incomplete ablation. If there is any residual, additional ablation could be performed to ensure complete ablation. （This video was generated by the interactive step-by-step sequence (TR, 3.1; TE, 1.57 ms; slice thickness, 10.0 mm; slice gap, 1.0 mm; and matrix scan, 96 × 94; total scan time: 0.202 s) and taken from monitor screen of MR by Nokia photo capture software）
